# Salivary IgG and IgA in newborn calves and the possible role in the assessment of passive immunity transfer

**DOI:** 10.3389/fvets.2024.1383379

**Published:** 2024-05-28

**Authors:** G. V. Berteselli, J. Filipe, A. Martelli, G. Vezzaro, E. Canali, P. Dall’Ara

**Affiliations:** ^1^Department of Veterinary Medicine and Animal Sciences, University of Milan, Via dell’Università, Lodi, Italy; ^2^Fiamenghi Domenico, Gianluigi, Claudio e Matteo S.S. Cascina San Giacomo, Strada San Giacomo, San Bassano, Cremona, Italy

**Keywords:** Brix refractometry, calf, ELISA, immunoglobulins, passive immunity transfer, saliva, welfare, ELISA test

## Abstract

**Introduction:**

The transfer of immunoglobulins from the mother to newborns is widely recognized as a critical event for safeguarding offspring against potentially life-threatening infectious diseases. Mainly for this reason, this study aimed to assess the concentrations of immunoglobulin G (IgG) and immunoglobulin A (IgA) in the saliva of newborn calves and explore its potential use for monitoring passive immunity transfer from cows to calves, as also to evaluate how colostrum intake affects serum and saliva IgG and IgA concentrations.

**Methods:**

The quality of colostrum samples was evaluated using an optical refractometer before administration to the calves. Saliva and blood samples from 24 calves were obtained at the day of birth (T0) and 2 days after (T2) for determination of serum concentrations of total protein by refractometer, IgG and IgA (both on serum and saliva) by ELISA test.

**Results:**

Positive correlations were observed between salivary IgA at T2 and salivary IgG at T2. A significant increase in both IgG and IgA levels in calf serum and saliva was noted. Salivary IgA levels can reflect salivary IgG levels.

**Discussion:**

These findings suggest the potential utility of IgA in monitoring passive immunity transfer, and do not exclude saliva as an alternative, practical, and non-invasive matrix for assessing passive immunity transfer.

## 1 Introduction

The transfer of immunoglobulins from the mother to the newborns is considered a crucial event for the protection of the offspring against dangerous and sometimes life-threatening infectious diseases. Due to their particular type of placenta, newly classified as cotyledonary synepitheliochorial (in the past syndesmochorial) (1), the passage of the Maternally Derived Antibodies (MDA) in cattle is only possible after birth through colostrum. For this reason, calves are born agammaglobulinemic and are entirely dependent on the intestinal absorption of colostrum MDA to receive their first humoral protection (2, 3). Colostrum is also a highly significant source of nutrients such as proteins, fats, carbohydrates, vitamins, minerals, and nutraceuticals, as well as maternal epithelial and immune cells (especially T and B cells and macrophages) that cross the intestinal barrier populating central and peripheral lymphoid tissues, then promoting, together with various bioactive compounds, the calf immune development and the calf growth (4–7).

Three major IgG subtypes can be distinguished in cattle: IgG1, IgG2 and the most recently discovered IgG3. IgG1 and IgG2 are the best characterized, with IgG1 being the most abundant in cow colostrum (up to 80–90% of the total IgG) (6). Besides IgG, also IgM are present in bovine colostrum (5–7%), where like in blood and milk they are the most abundant opsonic antibody class and are mainly involved in primary immune response. Finally, IgA are present in low quantities in bovine colostrum (2–5%) and serum as well, but are predominant in many secretions, especially in upper respiratory and gastrointestinal tracts (3, 6, 8–10). The newborn calf is able to absorb all three antibody classes, but IgA is partly re-secreted into the gut lumen, thus ensuring a robust and early local protection (7). Since both IgA and IgM have a shorter half-life (only 3–4 days) compared to IgG (21–28 days), they act as a primary defense in newborn calves providing protection for a brief period due to their relatively short life (8, 9).

Colostrum IgG are then the key component of newborn immunity, but IgG concentration is highly variable between cows (8, 11). A high-quality colostrum typically contains >50 mg/mL of IgG, while lower IgG concentrations indicate a low-quality colostrum (3, 8). Another factor that must be taken into serious consideration is the duration of intestinal absorption of these antibodies, as gut permeability decreases quickly in the first hours after birth, ending around the end of the second day (12).

Since IgG represents the most abundant antibody isotype in colostrum, their concentration is considered critical in assessing colostrum quality (3, 7, 8, 10, 12).

Calves are recommended to consume a volume of colostrum equivalent to 10 to 12% of their body weight (i.e., 3–4 L for a 30–40 kg Holstein calf), needing to receive 100 to 200 g of IgG within the first 6 h after birth to increase the likelihood of a successful passive transfer of immunity (3, 10). Therefore, ensuring that newborn calves receive an adequate amount of high-quality colostrum within the critical timeframe is widely recognized as a fundamental practice in calf rearing. This proactive approach significantly improves the success rate of transfer of passive immunity (2, 3, 13).

A deficiency of this latter, defined as Failure of Passive Transfer (FPT), increases the susceptibility of calves to infectious diseases and represents one of the main causes of mortality in calves during the first 3 weeks of life (3, 14, 15). In order to avoid this dangerous situation, many associations and experts in the field have proposed specific recommendations and the so-called “Three Q’s” rules as general guidelines for colostrum management and MDA transfer: “Quantity,” “Quickly,” and “Quality.” In addition, some authors suggest two other Q’s: “sQueakly clean” and “Quantifying passive transfer” (8, 16). The quality of colostrum can be easily assessed using optical Brix refractometer. This tool permits to approximate the percentage of total solids in a liquid. The obtained value represents the refractive index of a liquid. In detail, the implementation of optical refractometer requires an individual to peer into the instrument and determine the percentage Brix of the analyzed liquid by identifying a blue line on the scale (17).

The thresholds range of Brix refractometry suggested by literature varies from 18 to 22%, this latter corresponding to an IgG value of 50 mg/mL (17, 18). As suggested by Buczinski and Vandeweerd (13), the two cutoff points may be alternatively used to select good quality colostrum (Brix ≥22%) or to discard poor quality colostrum (Brix <18%). When the analyses provide an intermediate value, it is advisable to consider supplementation with high-quality colostrum to ensure an adequate immunity transfer from dam to calf. Implementing Brix refractometry on a farm is then easy and requires only a few drops of colostrum to obtain immediate results.

The passive transfer of immunity in calves is assessed by estimating serum IgG and total protein concentration (10, 19). For many years, radial immunodiffusion (RID) represented the only method able to directly measure and quantify IgG in bovine serum and colostrum. Over time, a specific ELISA method has been added alongside this classic and simple test. Both of these tests (RID and ELISA) represent direct testing methods to quantify the absolute IgG concentration in colostrum and blood, whereas indirect tests for both colostrum (e.g., Brix refractometry) and blood (e.g., Zinc Sulfate Turbidity) samples offer an approximate measurement of the immunoglobulin concentration. Alternatively, evaluating the blood levels of other components of colostrum, which are absorbed similarly to immunoglobulins (e.g., gamma-glutamyl-transferase activity) can provide an indication of the passive immunity level (10, 20–23).

To ensure a proper antibody transfer in calves, blood samples taken within 24 to 48 h after birth and colostrum intake are needed for a prompt analysis of IgG levels using direct tests (23). Unfortunately, the aforementioned methods are not directly applicable on farms and can be costly. It is highly beneficial to employ the same method to assess both the quality of colostrum and the success of passive immune transfer in the calf. In fact, the serum total protein evaluation using Brix refractometry has been recognized as a practical on-farm method to investigate the successful transfer of passive immunity in calves. Brix refractometry provides an estimate of the serum immunoglobulin concentration, as immunoglobulins represent a substantial proportion of the protein in neonatal calf serum (24, 25). A successful antibody transfer is indicated by a serum total protein concentration of >5.2 g/dL (equivalent to a serum IgG quantity of >10 mg/mL), while lower values are suggestive of FPT (10, 13, 16, 20, 23, 26–28). Since serum total proteins are correlated with immunoglobulin levels, their evaluation can reflect the effectiveness of colostrum absorption (20–23).

In order to evaluate the passive immunity, transfer in calves, a blood sampling is needed. This procedure can be quite invasive for the animal representing an ethical concern, and the presence of a veterinarian is required for its execution in some countries (e.g., Italy, Norway).

Saliva has proven to be a suitable immune matrix for evaluating different animal conditions (e.g., stress situation, immune system response, local protection) (29–32), and being its sampling non-invasive and stress-free and it does not require a veterinarian, it is easy to perform and preferable, when possible, compared to blood sampling (33, 34). In a recent pilot study of Johnsen et al. (35), saliva has been used for measuring total IgG levels in colostrated newborn calves in order to evaluate the success of passive immunity transfer, with interesting and promising results.

The aim of this study was to investigate the possibility to detect and quantify both IgG and IgA concentrations in the saliva of newborn calves, and its potential use for monitoring the correct transfer of passive immunity from cows to calves, and also to evaluate how colostrum intake affects serum and saliva IgG and IgA concentrations.

## 2 Materials and methods

### 2.1 Animals and management

The study was conducted from January to March 2023 in an Italian commercial dairy farm housing 1,012 lactating Holstein-Friesian dairy cows and 180 calves. Twenty-four Holstein-Friesian dairy heifer calves were recruited for the study. According to the farm’s protocol, newborn calves were allowed to remain with their mothers for a maximum of 1-h post-birth. Subsequently, the calves were transferred to individual pens. Within 6 h of birth, each calf received 3.5 liters of colostrum collected from the respective mother via bottle feeding. The quality of the initial colostrum was assessed using an optical Brix refractometer (RHB-32SG ATC, HHTEC, Heidelberg, Germany) calibrated within the range of 0–32% Brix. Colostrum samples were promptly assessed by placing a drop on the daylight plate of the refractometer. Colostrum was categorized as high quality if its Brix value exceeded 22%, in accordance with existing literature (13, 36).

In cases where colostrum did not meet the established quality threshold, calves were provided with high-quality colostrum sourced from another cow that had calved on the same day. Following the initial colostrum feeding, subsequent feedings consisted of colostrum or milk obtained from the mother’s subsequent milking. The quality of this subsequent colostrum was not evaluated, as it was deemed unnecessary at that stage. Calves were fed twice daily, with six feedings consisting of colostrum or milk obtained from their respective mothers. Following this period, calves were transitioned to a diet of milk powder (Sprayfo Royal). Additionally, the quality of initial colostrum sourced from both primiparous and pluriparous cows was compared to explore potential differences.

### 2.2 Saliva and serum sampling

In order to reduce the animal manipulation, both saliva and blood samples were collected at the same time from each calf at the day of birth (T0) and 2 days after (T2). Saliva samples were collected by placing a clamped synthetic polypropylene sponge into the calf’s mouth for chewing, as previously validated (46). Sponges were then placed in specialized collection devices (Salivette^®^, Sarstedt, Aktiengesellschaft & Co, Nümbrecht, Germany) and then centrifuged (3,000 g x 10′ at room temperature, RT). To minimize the contamination risk from previous milk meals, saliva samplings were performed prior to feeding. Blood samples were obtained via jugular vein puncture using 10 mL Vacutainer tubes and centrifuged (1,500 g x 10′ RT) after 30′ RT in order to obtain serum. Before storage, a drop of serum samples was analyzed using a standard optical refractometer (RETK-71, Tekcoplus Ltd., Hong Kong, China). This approach is used on farms to determine the adequacy of immune passive transfer. Refractometry provides an approximation of serum immunoglobulins concentration, because the immunoglobulins represent a great proportion of the serum protein in new-born calf (24, 37). Saliva and serum samples were thereafter stored at-20°C until use.

### 2.3 ELISA procedure

IgG1 and IgA concentrations in calves’ saliva and serum samples were analyzed by two ELISA sandwich kits (Bovine IgG1 ELISA Kit and Bovine IgA ELISA Kit, Bethyl Laboratories, Inc. Montgomery, Texas, respectively).

All samples have been appropriately diluted in the dilution buffer provided in the kit to achieve the expected saliva and serum concentration values falling within the calibration curves of the kits (IgG1 from 750 to 1.0 ng/mL – IgA from 1,000 to 1.37 ng/mL). In particular, samples were diluted differently based on the targeted antibody class: saliva 1:2,000 for IgG; serum 1:200,000 for IgG; saliva 1:2,000 for IgA; serum 1:2,000 for IgA. The diluted samples were then placed in the pre-coated IgG1/IgA 96-well microtiter plates (100 μL/well) in duplicate and incubated for 1 h RT. Plates were then washed 4 times (two rapid and two slow) with the wash buffer supplied by the kit before adding 100 μL/well of ready-to-use conjugate (anti-bovine IgG1 or anti-bovine IgA biotinylated detection antibodies), incubated 1 h RT. After washing, a ready-to-use streptavidin-conjugated horseradish peroxidase was then added (100 μL/well) to catalyze the colorimetric reaction with the substrate-chromogen substrate (H2O2 and TMB; 3,3′, 5,5′ tetramethylbenzidine) added after a final series of washes, leaving it 15 min in the dark RT. The reaction was stopped adding 100 μL/well of stop solution (sulfuric acid 0.18 M) and the plates were then read at 450 nm with an ELISA microplate reader (Titertek Multiskan, Labsystem), expressing the result as O.D. (Optical Density).

### 2.4 Statistical analysis

Statistical analysis was performed using Graph Pad Prism 9 software (GraphPad Software, La Jolla, CA, United States). To assess the normality of the data, the D’Agostino-Pearson normality test, Shapiro–Wilk test, and Kolmogorov–Smirnov test have been employed. Subsequently, based on the nature of the samples under consideration, various statistical tests were applied, including Spearman correlation, T-test, Mann–Whitney test, paired T-test, and Wilcoxon test.

Statistical significance was established for *p*-values <0.05, while trends were noted for p-values between 0.05 and 0.1. This comprehensive approach ensures a robust evaluation of the data, employing both parametric and non-parametric tests as appropriate.

## 3 Results

The refractometry on colostrum given to calves in this study showed an average of Brix value of 27% (min.25% - max 30%), which exceeded the cutoff of 22%. This indicates a high-quality colostrum. On calves’ serum the average value of total proteins was 6.5 mg/mL (min 5 mg/mL – max 8 mg/mL), suggesting an adequate transfer of passive immunity.

A preliminary analysis was conducted using the Spearman correlation, where all variables were tested, yielding significant results. Positive and statistically significant correlations were found between salivary IgA at T2 and salivary IgG at T2 (0.640; *p*-value = 0.001), and between serum IgA at T2 and serum proteins at T2 (0.745, *p*-value<0.001). There is also a positive trend toward significance between salivary IgA at T0 and salivary IgG at T0 (0.376, *p*-value = 0.064), and between salivary IgA at T0 and salivary IgA at T2 (0.389, *p*-value = 0.055; Table 1).

**Table 1:** Correlations recorded for immunological parameters (Spearman correlation) considering statistically significant values at *p* < 0.05 and tendencies at *p* < 0.1.

Parameters		Rho (Spearman)	*p*-value
salivary IgA at T0	salivary IgG at T0	0.376	0.064
	salivary IgA at T2	0.389	0.055
salivary IgA at T2	salivary IgG at T2	0.640	0.001
serum IgA at T2	serum proteins at T2	0.745	<0.001

### 3.1 Saliva

**Figure 1:**
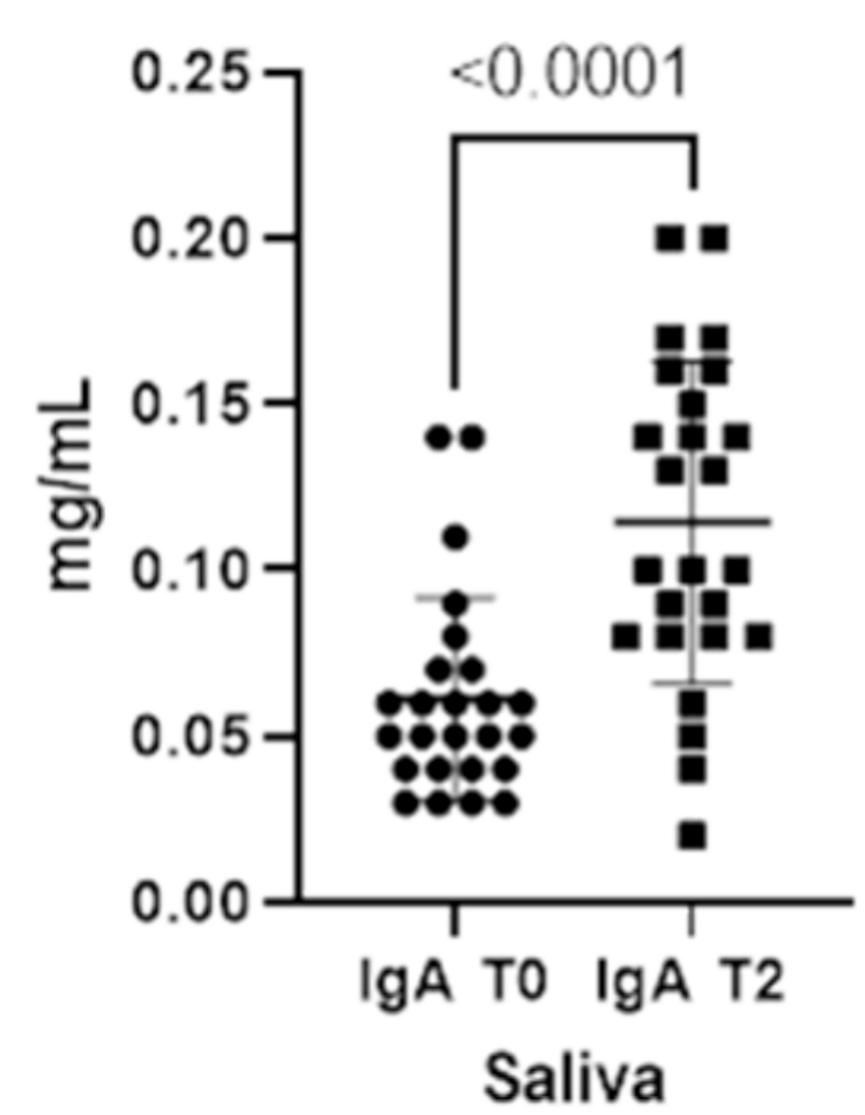
Immunoglobulins (IgA) concentrations present in saliva at two different timepoints (T0 and T2).

**Figure 2:**
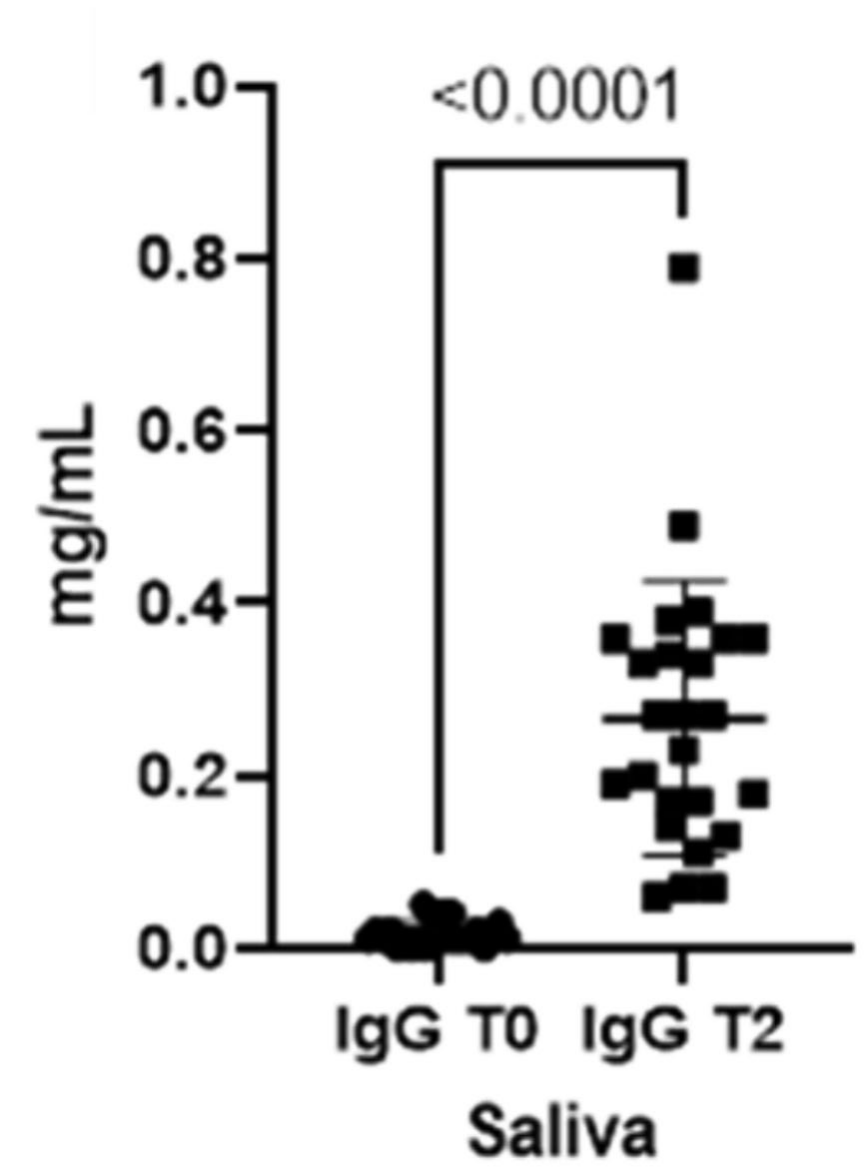
Immunoglobulins (IgG) concentrations present in saliva at two different timepoints (T0 and T2).

When comparing both IgA and IgG concentrations in saliva at the two different timepoints, we could verify a statistically significant increment for both immunoglobulins from T0 to T2 (Figures 1, 2).

### 3.2 Serum

**Figure 3:**
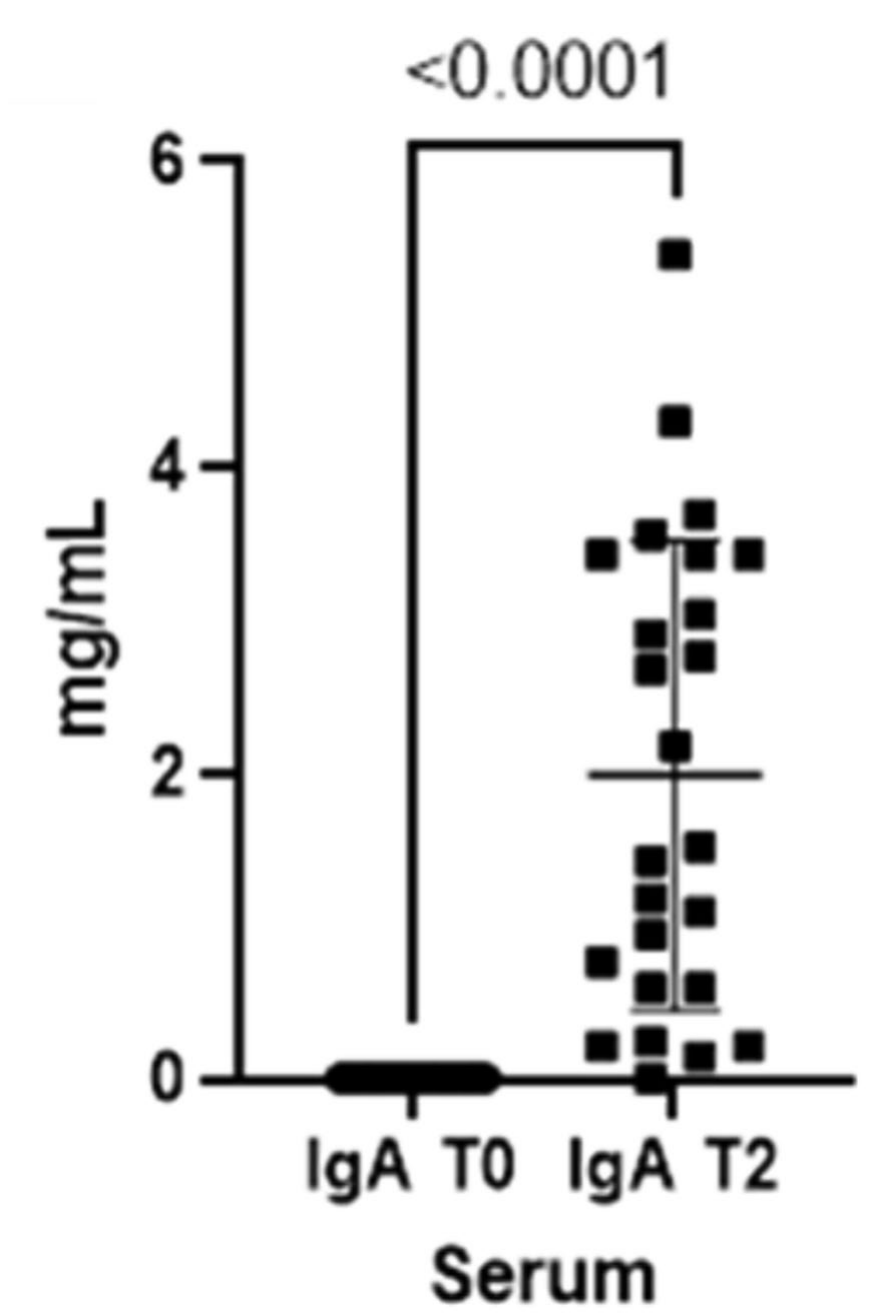
Immunoglobulins (IgA) concentrations present in the blood at two different timepoints (T0 and T2).

**Figure 4:**
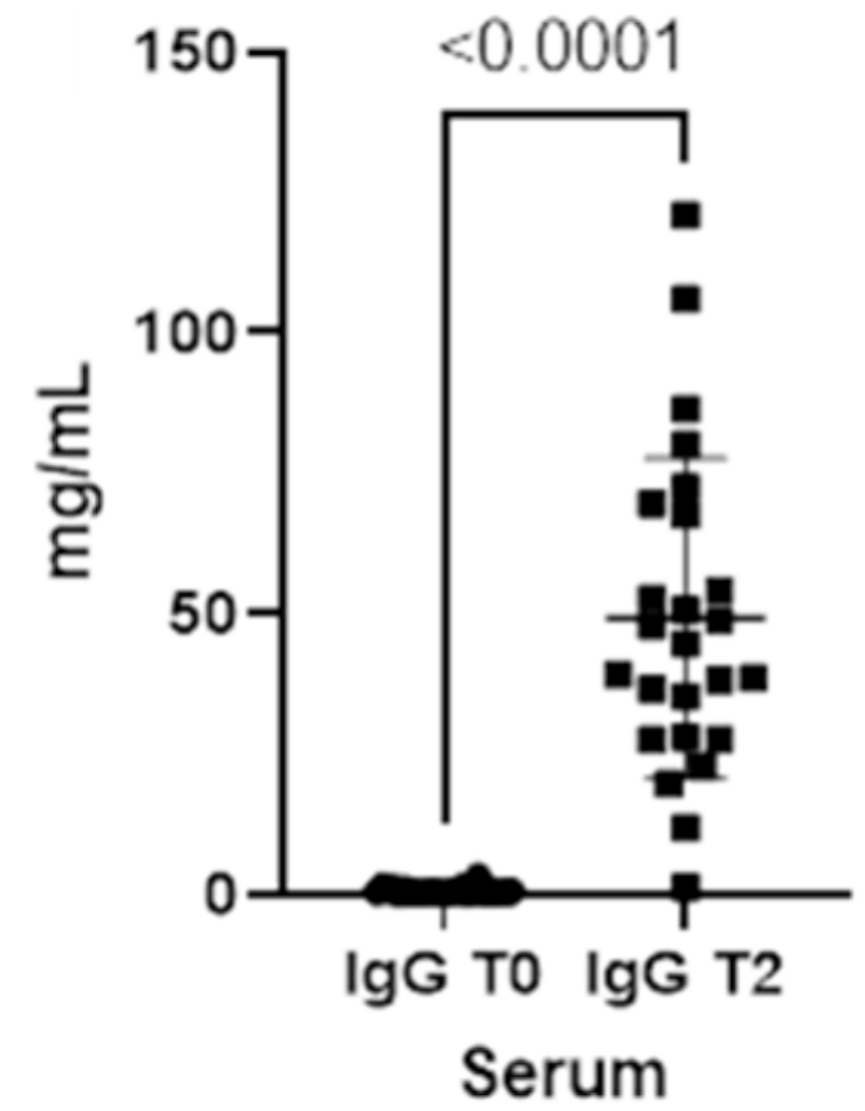
Immunoglobulins (IgG) concentrations present in the blood at two different timepoints (T0 and T2).

As already seen for the saliva, the concentrations for both immunoglobulins also increase in the blood from T0 to T2 (Figures 3, 4).

**Figure 5:**
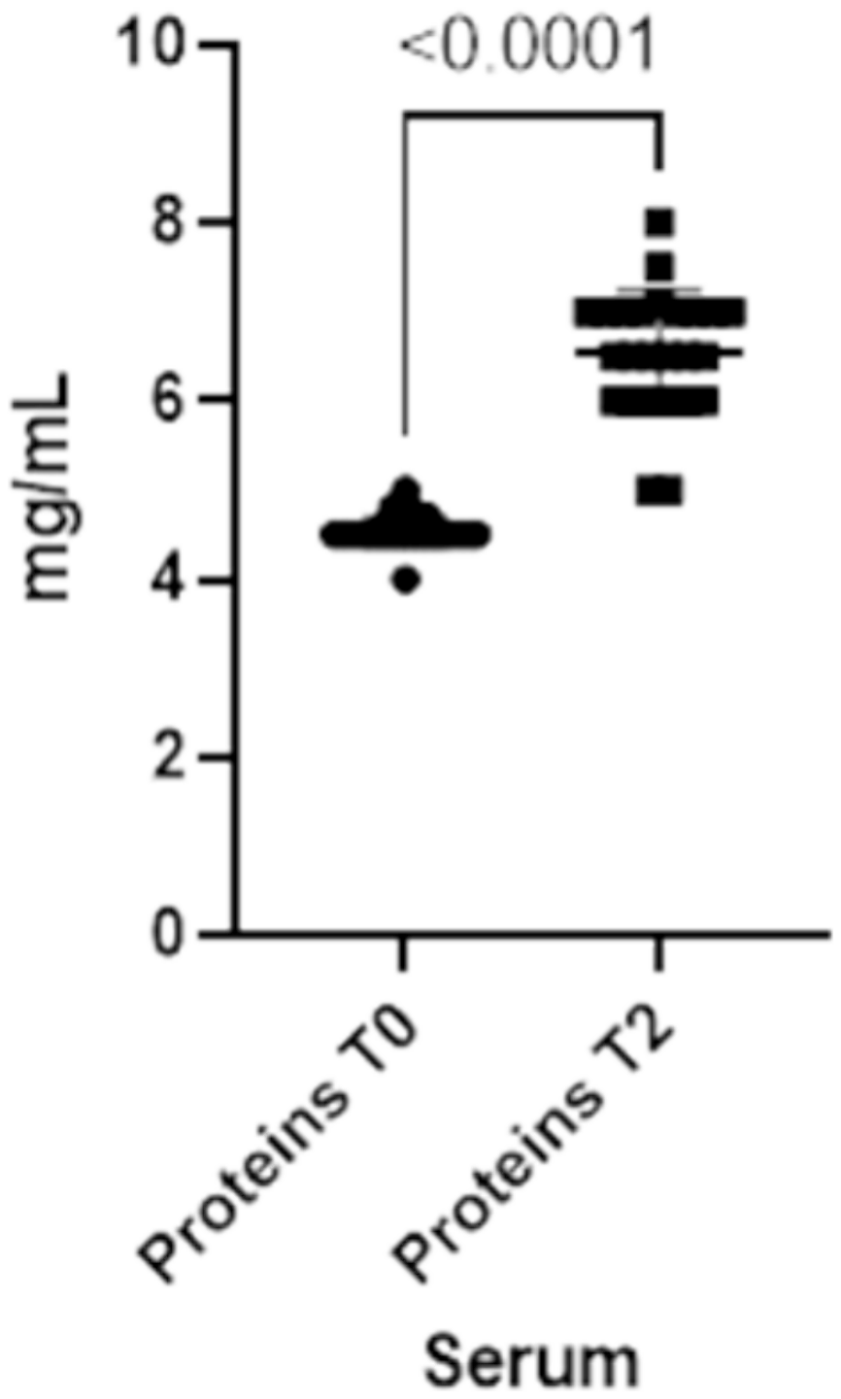
Total proteins concentrations present in the blood at two different timepoints (T0 and T2).

Finally, total serum proteins increase at T2 (*p*-value<0.0001), reflecting the changes in both IgA and IgG in serum (Figure 5).

### 3.3 Saliva vs. serum

The results indicate that serum IgG levels are consistently higher when compared with salivary ones, at both T0 (*p*-value<0.0001) and T2 (*p*-value<0.0001), and salivary IgA are higher than serum IgA at T0 (*p*-value<0.0001). When compared with T0, serum and salivary IgA levels are higher at T2, as well as salivary and serum IgG at T2. Additionally, our data demonstrated that serum IgA is higher than salivary IgA at T2 (*p*-value<0.0001).

### 3.4 Primiparous vs. pluriparous animals

**Figure 6:**
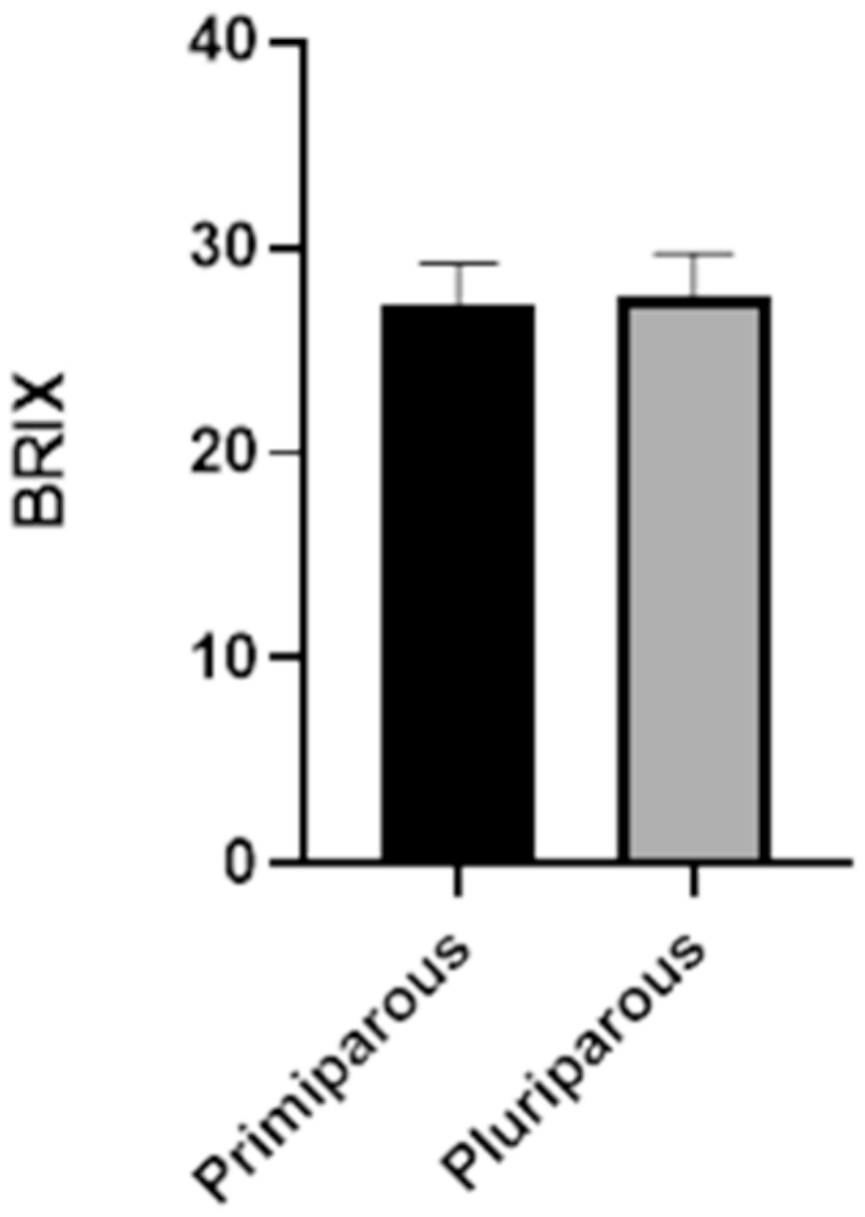
Comparison of BRIX values of primiparous versus pluriparous animals.

Lastly, it was determined that there were no differences between primiparous and pluriparous individuals in the colostrum Brix value (Figure 6).

## 4 Discussion

The quality of colostrum depends mostly on the appropriate concentration of immunoglobulins, with the IgG being the most abundant ones in cows’ colostrum. Specifically, the IgG1 are transferred in substantial quantities to the udder and into the secretions, as IgA do in many other species. However, IgA presence in cattle is also evident in secretions such as saliva, tears, bronchial secretions, and also colostrum, playing a main role in local immune defenses (6, 9). Colostrum IgG levels can be indirectly measured on-farm using refractometry technology. Some studies have suggested a cut-off point of 22% Brix to identify a good colostrum, representing a concentration of 50 mg of IgG/mL (3, 13, 16, 20, 23). In our study a mean Brix value of 27% was obtained, indicating high colostrum quality in the recruited farm without discernible differences between primiparous and multiparous cows (Figure 6). Consequently, the practice of automatically discarding colostrum from first-calf heifers should be discouraged, at least without a previous colostrum quality analysis (3). An optimal colostrum intake is crucial for calf health, although it is not the sole factor to consider (38). Assessing passive immunity transfer status in calves is essential for detecting failure of passive transfer of immunity (FPT), and to implement appropriate treatment management plans (i.e., colostrum supplementation), reduce mortality, and improve animal welfare (3, 38).

Serum total proteins concentration can serve as an indirect indicator for assessing passive immunity transfer, since it is primarily influenced by colostrum antibody absorption in the gut (3, 13, 24, 38). Refractometry analysis of calf serum total proteins is considered a useful method for determining the adequacy of passive immunity transfer, providing an estimation of IgG concentration (39). The use of refractometer has undeniable advantages as it is a convenient, rapid, inexpensive, and portable tool easy to use on the farm. However, for the assessment of IgG transfer, a blood sample is needed, and this can be highly stressful and painful for the calf. Moreover, in some countries (e.g., Italy, Norway) only veterinarians can perform this medical practice (35). Conversely, saliva sampling does not require the veterinarian intervention, and it could be a minimally invasive and simple approach to investigate the passive immunity transfer in the calves (30, 32, 35). However, very few studies have explored the concentration of salivary IgG levels and its possible applicability for this purpose (11). Moreover, to the authors’ knowledge, no study had explored serum and salivary IgA in neonatal calves, and their possible role in evaluating the passive immunity transfer.

The preliminary results of the present study proved the presence of detectable values of IgG and IgA in calf’s saliva in pre and post-colostrum intake. A positive correlation between salivary IgA and salivary IgG concentrations post-colostrum intake (0.640; *p*-value = 0.001) was found, suggesting that salivary IgA can reflect salivary IgG levels. Similarly, a positive correlation between serum IgA and serum proteins post-colostrum intake was observed (0.745; *p*-value<0.001). While no significant correlation was observed between serum and saliva parameters, the findings of the present study underscore the impact of colostrum intake on immunoglobulin concentrations. This resulted in an elevation of both IgA and IgG levels not only in the serum but also in the saliva of the calves. These results do not preclude the potential utility of saliva in assessing passive immunity transfer. The significance of this discovery cannot be overstated, as it reveals a direct influence of colostrum intake on both systemic and local immunity. This suggests a synergy between systemic and local immune responses in newborn calves.

In fact, colostrum contains key components such as casein, lactoferrin, immune cells (i.e., macrophages, T and B lymphocytes), and other bioactive elements that contribute to development and maturation of the calf’s immune system (6, 11, 40). The notable rise in immunoglobulin concentrations in saliva following colostrum intake is likely attributed to passive immunity transfer: since the increase occurs in a very short time (2 days), it cannot be attributed to active antigenic stimulation of the neonate’s immune system (9, 35). Additionally, measures were taken to prevent contamination of saliva samples by colostrum, with sampling conducted away from colostrum intake (2 h before both time-points). As suggested by Johansen et al. (35), the analysis of pre-colostral saliva was performed and revealed detectable levels of IgG in newborn calves. It is plausible that IgG1 and IgA present in cow vaginal and cervix secretions may have come into contact with the calf’s mouth during calving (9). Despite calves’ precolostral serum typically lacking IgG, detectable IgG1 levels were found in this study. This could be due to the ability of bovine fetuses to respond to antigenic stimuli (especially viruses such as rotavirus, parvovirus, and parainfluenza 3 virus) even before birth (9). Moreover, natural antibodies represent a potentially important humoral component of the newborn innate immune system, mostly represented by IgM and some IgG and IgA. These are antibodies present in healthy animals without (known or deliberate) antigenic stimulation, providing immediate, early and broad protection against pathogens (6, 41, 42). The presence of natural antibodies in the calves before the colostrum intake was confirmed by a study of Maysary et al. (43).

Refractometry is a convenient tool for on-farm assessment of passive immunity transfer using calf serum, however it has the limitation of not being able to detect small concentration differences, leading to potential negative effects in identifying FPT (44). The results of this study support the use of ELISA as a direct and more accurate measurement of immunoglobulins in both blood and saliva. The role of saliva as a possible alternative matrix in the assessment of immunity in calves should be further investigated based on these preliminary results, since both salivary IgG and IgA concentrations seem influenced by the colostrum intake. In addition, saliva analysis permits the detection of both IgG and IgA, providing comprehensive insights into the animal’s immunity status.

The potential development of a rapid ELISA test kit for quantifying IgG and/or IgA could assist in situations where there are uncertainties about failure of passive transfer or when refractometer values are borderline, as already done and available for the foal (45). So, the future development of an in-clinics ELISA kit for assessing salivary immunoglobulins could offer farmers a further tool for managing calf colostrum and detecting FPT, ultimately improving calf health and welfare.

Finally, as previously mentioned, refractometer is an easy, quick, and good on-farm tool to assess the transfer of passive immunity using calf’s serum (27), while ELISA analysis is not suitable for on-field use due to higher costs and the lack of immediate results. Future investigations should aim to assess the potential application of Brix for quantifying salivary immunoglobulins, although preliminary results of Johnsen et al. (35) did not support the usefulness of this approach to assess calf passive immunity transfer, especially for the possibility to bypass both ethical and feasibility barriers related to blood sampling, and costs and time related challenges with ELISA. Another very interesting study should be the salivary IgM quantification in pre- and post-colostrum calves in order to assess their role in the passive immunity transfer and, since they are quickly produced after a first antigenic stimulation.

The findings of this preliminary study should be interpreted within the context of its limitations, particularly in terms of sampling and measurement. To validate the promising results regarding saliva’s potential as an alternative biological matrix for assessing passive immune transfer, a larger sample size encompassing a greater number of calves from diverse farms is necessary. Additionally, the absence of a negative control group in this study warrants attention. All calves received high-quality colostrum, and ethical considerations precluded deliberately withholding colostrum from a control group, as it would significantly impact animal welfare and survival.

Consequently, it was not feasible to establish a definitive saliva level cut-off in this study. To address these limitations, future research endeavors could focus on expanding the sample size and comparing salivary immunoglobulin levels among groups of calves fed varying qualities of colostrum, including both high and low quality.

It is important to note that the data for this study were obtained from a commercial dairy farm, so standard procedures for colostrum administration and calves’ management in place on the farm were considered in the setting of the study.

## 5 Conclusion

The correct transfer of passive immunity from dam to calf should be assessed routinely on farm in order to limit the susceptibility of infectious diseases and mortality, and to improve the short- and long-term health and welfare. The evaluation of calf’s serum total protein by refractometry has proven to be a reliable and convenient on-farm method to determinate the adequate passive immunity transfer, giving an indirect estimation of IgG serum concentration. However, this method requires blood sampling, which needs specialized intervention (e.g., veterinarian) in some cases, and animal constraint, and can be a source of pain and stress. Therefore, measuring immunoglobulins in saliva could be a potential simpler, and non-invasive method to determine the transfer of passive immunity. ELISA test permits a direct immunoglobulins quantification and class (IgG and/or IgA) identification. Nevertheless, the high cost, the requirement for a laboratory to analyze samples, and the longer timeframes to achieve results make it impractical for on-farm use. The use of refractometry to assess the calf’s saliva immunoglobulin concentration thereby avoiding ethical and economic concerns, along with the development of a rapid, field-portable ELISA kit should be considered useful tool for improved colostrum management in calves. These methods could also aid in identifying partial or total Failure of Passive Transfer (FPT) or investigating borderline cases.

## Data availability statement

The raw data supporting the conclusions of this article will be made available by the authors, without undue reservation.

## Ethics statement

The animal studies were approved by Animal Welfare Committee of the University of Milan (OPBA_140_2022; December 6, 2022). The studies were conducted in accordance with the local legislation and institutional requirements. Written informed consent was obtained from the owners for the participation of their animals in this study.

## Author contributions

GB: Conceptualization, Data curation, Formal analysis, Investigation, Methodology, Validation, Writing – original draft, Writing – review & editing. JF: Data curation, Formal analysis, Investigation, Methodology, Software, Validation, Writing – original draft, Writing – review & editing. AM: Investigation, Resources, Writing – review & editing. GV: Investigation, Writing – review & editing. EC: Conceptualization, Funding acquisition, Project administration, Supervision, Writing – review & editing. PD’A: Conceptualization, Data curation, Formal analysis, Investigation, Methodology, Project administration, Supervision, Validation, Writing – original draft, Writing – review & editing.
